# Association between De Ritis ratio (aspartate aminotransferase/alanine aminotransferase) and oncological outcomes in bladder cancer patients after radical cystectomy

**DOI:** 10.1186/s12894-019-0439-7

**Published:** 2019-01-24

**Authors:** Yun-Sok Ha, Sang Won Kim, So Young Chun, Jae-Wook Chung, Seock Hwan Choi, Jun Nyung Lee, Bum Soo Kim, Hyun Tae Kim, Eun Sang Yoo, Tae Gyun Kwon, Won Tae Kim, Wun-Jae Kim, Tae-Hwan Kim

**Affiliations:** 1 Department of Urology, School of Medicine, Kyungpook National University, Daegu, South Korea; 2 Department of Urology, School of Medicine, Kyungpook National University, Kyungpook National University Hospital, Daegu, South Korea; 3 Department of Urology, Chungbuk National University College of Medicine, Cheongju, South Korea

**Keywords:** Bladder cancer, Prognosis, Survival, De Ritis ratio

## Abstract

**Background:**

New biological prognostic predictors have been studied; however, some factors have limited clinical application due to tissue-specific expression and high cost. There is the need for a promising predictive factor that is simple to detect and that is closely linked to oncological outcomes in patients with urothelial bladder cancer (BC) who have undergone radical cystectomy (RC). Therefore, we investigated the clinical prognostic value of the preoperative De Ritis ratio (aspartate aminotransferase/alanine aminotransferase) on oncological outcomes in patients with urothelial BC after RC.

**Methods:**

We retrospectively evaluated clinicopathological data of 118 patients with non-metastatic urothelial BC after RC between 2008 and 2013 at a single center. The association between the De Ritis ratio and clinicopathological findings was assessed. The potential prognostic value of the De Ritis ratio was analyzed using the Kaplan-Meier method, and multivariate Cox analyses were performed to identify the independent predictors of metastasis-free survival, cancer-specific survival, and overall survival.

**Results:**

According to the receiver operating curve of the De Ritis ratio for metastasis, we stratified the patients into 2 groups using a threshold of 1.3. A high De Ritis ratio was more likely to be associated with old age and the female sex. Kaplan-Meier estimates revealed that patients with a high De Ritis ratio had inferior metastasis-free survival, cancer-specific survival, and overall survival outcomes (*P* = 0.012, 0.024, and 0.022, respectively). Multivariate analysis revealed that a high De Ritis ratio was an independent prognostic factor for metastasis (hazard ratio [HR], 2.389; 95% confidence interval [CI], 1.161–4.914; *P* = 0.018), cancer-related death (HR, 2.755; 95% CI, 1.214–6.249; *P* = 0.015), and overall death (HR, 2.761; 95% CI, 1.257–6.067; *P* = 0.011).

**Conclusions:**

An elevated De Ritis ratio was significantly associated with worse prognosis in patients who underwent RC for urothelial BC. This ratio might further improve the predictive accuracy for prognosis in BC.

## Background

Urothelial carcinoma typically occurs in the urinary system: the kidney, urinary bladder, and accessory organs [1]. It is the most common type of bladder cancer (BC) and cancer of the ureters, urethra, and urachus. BC is the second most common malignancy of the genitourinary tract, and shows a male predominance in Korea; it has the seventh highest incidence in men [2]. An estimated total of 3824 new BC cases and 1412 BC-related deaths were expected to occur in Korea in 2016 [3]. Among newly diagnosed patients, approximately 70–80% present with non-muscle-invasive BC (NMIBC). NMIBC is typically managed by transurethral tumor resection, a minimally invasive surgical procedure. The prognosis of NMIBC patients can be favorable if the disease has not progressed to muscle-invasive BC (MIBC). However, NMIBC eventually progresses to MIBC in approximately 30% of patients. Radical cystectomy (RC) with pelvic lymph node dissection (PLND) is the customary treatment option for local MIBC. RC with PLND is sometimes used to treat NMIBC, including Bacille Calmette-Guerin (BCG)-refractory cases and high-grade tumors. Nonetheless, approximately 50% of patients experience a relapse within 2 years. The 3-year survival rate is less than 50% [4]. Neoadjuvant chemotherapy before RC has been recognized as a treatment to improve cancer-specific survival (CSS) rates. These results provided evidence for neoadjuvant chemotherapy [5, 6]. However, eligible patients are difficult to identify because of the poor prognostic value of the current clinical staging system, resulting in its underuse [7]. Therefore, a preoperative prognostic factor capable of adequately stratifying patients for optimal preoperative management is needed.

In most clinical fields, alanine aminotransferase (ALT) and aspartate aminotransferase (AST) are the most utilized liver enzymes. In 1957, the ratio of the serum activities of AST and ALT was initially described by De Ritis, and has been known as the De Ritis ratio (AST/ALT) [8]. Cancer and non-cancerous tissues generated these enzymes and they have been stated as important prognosticators in lots of malignant tumors. These include multiple myeloma, colonic, pancreatic, renal cell carcinoma (RCC), and upper tract urothelial cancer (UTUC) [9–13]. For example, Bezan et al. reported that the preoperative De Ritis ratio was an independent prognostic factor in patients with non-metastatic RCC [12]. The De Ritis ratio is hypothesized to be associated with increased anaerobic glycolysis, a process known as the Warburg effect. Due to the fact that urothelial carcinoma was also reported to be related to glucose metabolism, we hypothesized that the De Ritis ratio might also have a prognostic role in BC [14, 15]. To the best of our knowledge, the prognostic value of the De Ritis ratio has not been evaluated in patients with BC. Therefore, we aimed to evaluate the prognostic value of the De Ritis ratio in patients who underwent RC for BC.

## Methods

Between August 2008 and May 2013, 118 patients with non-metastatic urothelial BC underwent RC at our hospital following Institutional Review Board approval (approval number: KNUMC 2016–05-021). Before RC, all the patients underwent transurethral resection of bladder tumors (TUR-BTs). After histopathological analyses and checking up the images, we carried out RC. The most of patients underwent RC were MIBC free of remote metastasis. Additionally, recurrent multifocal superficial refractory tumor, repeated transurethral resection, and BCG-resistant carcinoma in situ (CIS) are also indicators. We excluded the pateitns with previous pelvic radiation, clinical stage M1, prior combination surgery, and patients with chronic liver disease (hepatitis, liver cirrhosis, and severe fatty liver disease) including hepatitis B or C virus carriers. Open RC was performed through a midline incision in the typical manner [16, 17]. Robot-assisted RC was performed using the same surgical procedure, as reported by Menon et al. [18, 19]. The 2010 American Joint Committee on Cancer (AJCC) TNM staging system for BC was used for clinical T stage. [20]. Histologic grades were determined according to the 2004 World Health Organization (WHO) classification system [21]. Measurements of AST and ALT were routinely included in our preoperative workup and were performed before RC. Cisplastin-based chemotherapy was performed for at least 4 cycles in patients with good performance status among those with pT3, pT4, and node-positive disease. We used the published guidelines that apply to each patient for management and follow-up. [22].

**Fig. 1:**
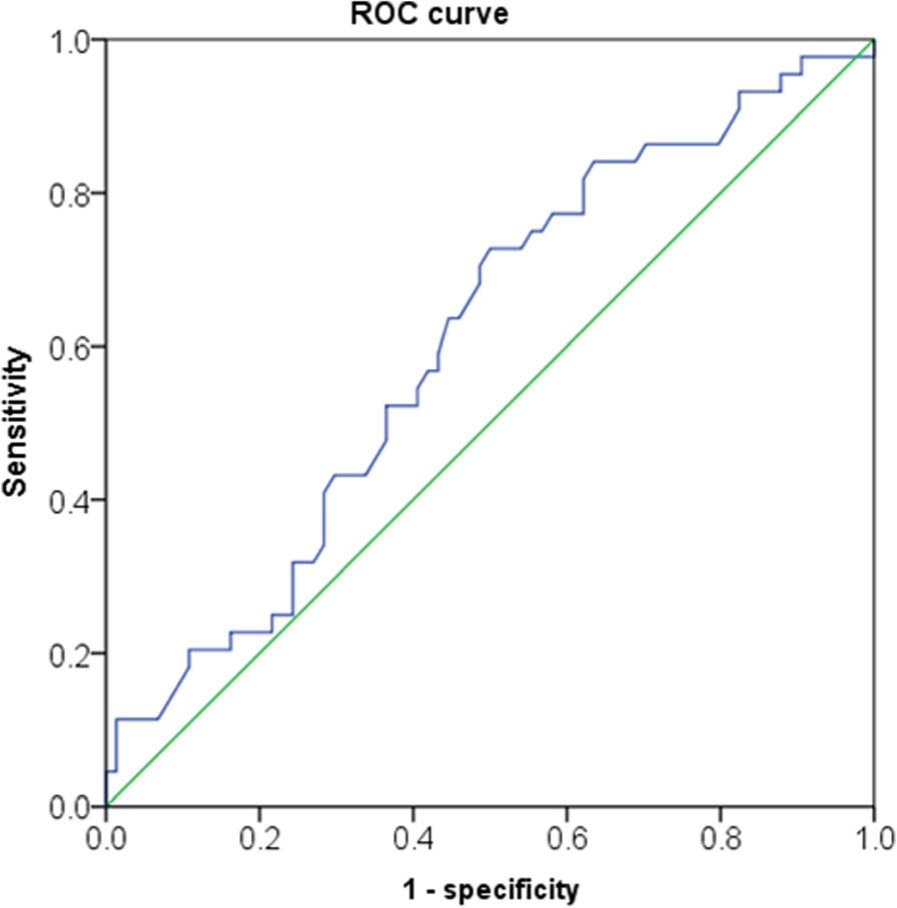
Receiver operating characteristics (ROC) curve of pretreatment De Ritis ratio

To settle the ideal cutoff level, the receiver-operating characteristic (ROC) curve of the De Ritis ratio for metastasis (44 metastasis vs. 74 non-metastasis) was used. An optimal cutoff value of 1.3 was based on a maximal Youden index at this value. The area under the curve of the De Ritis ratio was 0.606 (Fig. 1). Subsequently, the high De Ritis ratio cohort was defined patients with De Ritis ratios ≥1.3 and the others (De Ritis ratio < 1.3) were allocated to the low De Ritis ratio cohort. Student’s t-test and chi-square test were used to compare the clinicopathological features stratification according to De Ritis ratio. The survival spreads, including metastasis free survival (MFS), CSS and overall survival (OS), were assessed by the Kaplan-Meier method. Comparison of survival distributions was performed by a log-rank test between two cohorts. Factors independently associated with MFS, CSS, and OS were determined using a multivariate Cox proportional hazard regression model, with hazard ratios (HRs) and 95% confidence intervals (CIs) calculated for each factor. Differences with p<0.05 were considered statistically significant. IBM SPSS ver. 18.0 (IBM Co., Armonk, NY, USA) was used for statistical analyses.

## Results

Among the 118 patients, 49 had low De Ritis ratios and 69 had high De Ritis ratios. Their median age was 69 years (interquartile range, 60–74). Patients with high De Ritis ratios were significantly older and predominantly women, but were otherwise similar to patients with low De Ritis ratios with respect to body mass index, American Society of Anesthesiologists score, receipt of BCG instillation and neoadjuvant chemotherapy, clinical stage at the time of the latest TUR-BT, and presence of CIS at the time of the last TUR-BT (Table 1).

**Table 1:** Patient demographics and preoperative characteristics

Parameters	Low De Ritis ratio (< 1.3, *N* = 49)	High De Ritis ratio (≥1.3, *N* = 69)	*P*
Age, y			0.029
< 70	32 (65.3)	31 (44.9)	
≥70	17 (34.7)	38 (55.1)	
Gender			0.032
Male	45 (91.8)	53 (76.8)	
Female	4 (8.2)	16 (23.2)	
BMI (kg/m^2^, ±SD)	22.97 ± 3.28	22.16 ± 2.94	0.156
ASA classification			0.617
1	8 (16.3)	9 (13.0)	
≥2	41 (83.7)	60 (87.0)	
Clinical stage at latest TUR-BT			0.609
≤T1	19 (38.8)	30 (43.5)	
≥T2	30 (61.2)	39 (56.5)	
Presence of CIS at last TUR-BT			0.221
No	47 (95.9)	62 (89.9)	
Yes	2 (4.1)	7 (10.1)	
BCG instillation history			0.678
No	45 (91.8)	60 (89.6)	
Yes	4 (8.2)	7 (10.4)	
Neoadjuvant chemotherapy			0.92
No	40 (81.6)	56 (82.4)	
Yes	9 (18.4)	12 (17.6)	

*BMI* body mass index, *BCG* Bacille Calmette-Guerin, *ASA* American Society of Anesthesiologists, *TUR-BT* transurethral tumor resection of bladder tumor, *CIS* carcinoma in situ

Comparisons of clinicopathological variables according to De Ritis ratios are summarized in Table 2. Most clinicopathological parameters, including pathological and histological grade, lymph node involvement, and lymphovascular invasion, did not differ significantly between the two groups.

**Table 2:** Comparison of clinicopathological variables according to De Ritis ratio in 118 patients who underwent radical cystectomy

Parameters	Low De Ritis ratio (< 1.3, *N* = 49)	High De Ritis ratio (≥1.3, *N* = 69)	*P*
Pathologic stage			0.75
T0, Tis, Ta	4 (8.2)	6 (8.7)	
T1	11 (22.4)	17 (24.6)	
T2	16 (32.7)	15 (21.7)	
T3	12 (24.5)	22 (31.9)	
T4	6 (12.2)	9 (13.0)	
Histologic grade			0.072
Low	4 (8.2)	1 (1.4)	
High	45 (91.8)	68 (98.6)	
Lymph node involvement			0.777
No	38 (77.6)	55 (79.7)	
Yes	11 (22.4)	14 (20.3)	
Lymphovascular invasion			0.465
No	41 (83.7)	54 (78.3)	
Yes	8 (16.3)	15 (21.7)	
Median follow-up periods (months, range)	40 (6.5–83.3)	31.8 (4.3–95.3)	0.089
Metastasis			0.015
No	37 (75.5)	37 (53.6)	
Yes	12 (24.5)	32 (46.4)	
Cancer-related death			0.045
No	39 (79.6)	43 (62.3)	
Yes	10 (20.4)	26 (37.7)	
Overall death			0.039
No	38 (77.6)	41 (59.4)	
Yes	11 (22.4)	28 (40.6)	

**Fig. 2:**
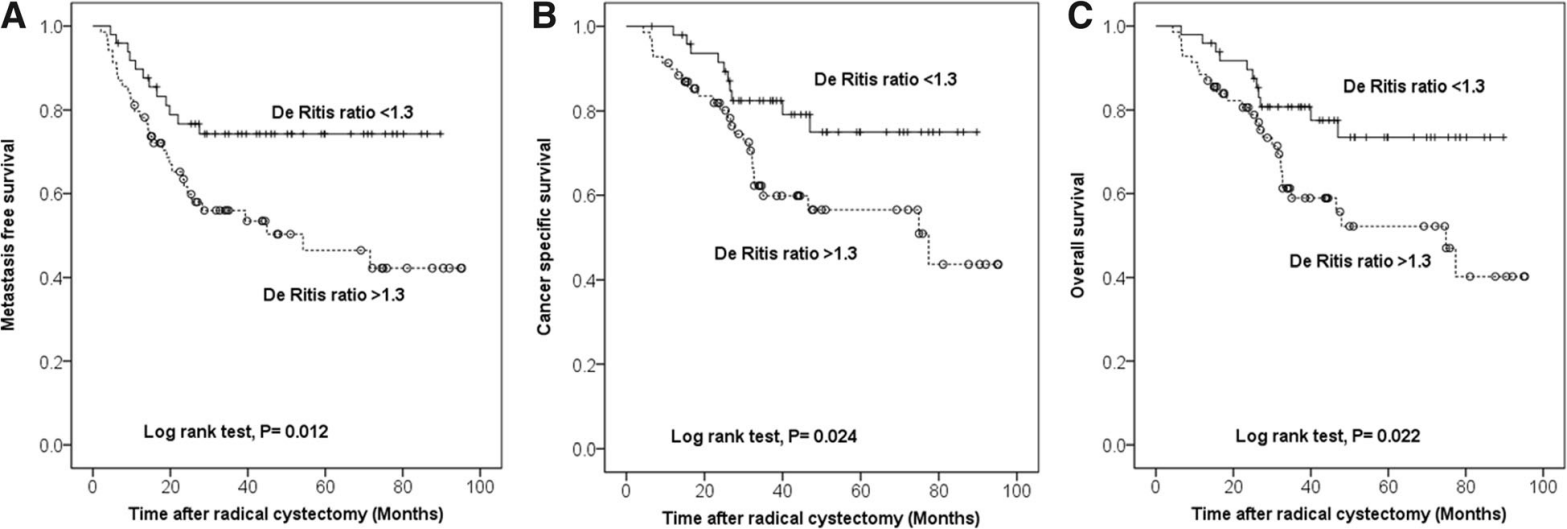
Kaplan–Meier curves predicting metastasis-free survival (**a**), cancer-specific survival (**b**), and overall survival (**c**) according to the De Ritis ratio

The median follow-up duration was 34.1 months, during which 44 patients showed metastasis and 39 patients died, including 36 who died due to BC. Notably, patients with high De Ritis ratios had significantly inferior MFS (53.6% vs. 75.5%; *P* = 0.015), CSS (62.3% vs. 79.6%; *P* = 0.045), and OS (59.4% vs. 77.6%; *P* = 0.039) rates than those with low De Ritis ratios (Table 2). The Kaplan-Meier analyses showed significantly inferior survival outcomes for MFS (*P* = 0.012), CSS (*P* = 0.024), and OS (*P* = 0.022) in patients with high De Ritis ratios (Fig. 2). Moreover, the De Ritis ratio was found to be independently associated with metastasis (HR, 2.389; *P* = 0.018), cancer-related death (HR, 2.755; *P* = 0.015), and overall death (HR, 2.761; *P* = 0.001) on multivariable analysis (Table 3). To confirm these results, we performed additional analyses. The De Ritis ratio was tested as a continuous variable in another multivariate Cox regression model. The preoperative De Ritis ratio as a continuous variable (*P* = 0.008, *P* = 0.003, and *P* = 0.002, respectively) and pathological T stage (*P* = 0.008, *P* < 0.001, and *P* < 0.001, respectively) were independent predictors of metastasis, cancer-related death, and overall death (Table 3).

**Table 3:** Multivariate Cox regression analysis of factors predictive of prognosis in bladder cancer after radical cystectomy

Parameters	HR	95% CI	*P*	HR	95% CI	*P*	Parameters	HR
Multivariate Cox proportional analysis to metastasis								
Age (< 70 vs. ≥70)	0.994	0.959	1.030	0.725	0.998	0.964	1.033	0.894
Gender (male vs. female)	0.825	0.354	1.923	0.655	0.745	0.288	1.928	0.544
BMI	0.972	0.867	1.088	0.618	0.957	0.854	1.073	0.450
Pathologic T stage	1.667	1.234	2.251	0.001	1.776	1.296	2.434	< 0.001
Lymph node involvement	1.727	0.895	3.335	0.103	1.816	0.942	3.501	0.075
Grade	0.640	0.078	5.249	0.678	0.549	0.065	4.629	0.582
De Ritis ratio (Low vs. high)	2.389	1.161	4.914	0.018				
De Ritis ratio (Continuous)					1.582	1.129	2.219	0.008
Multivariate Cox proportional analysis of cancer-related death								
Age (< 70 vs. ≥70)	0.996	0.959	1.035	0.834	1.000	0.964	1.038	0.988
Gender (male vs. female)	0.488	0.164	1.448	0.196	0.363	0.096	1.377	0.136
BMI	0.941	0.825	1.073	0.361	0.919	0.807	1.046	0.200
Pathologic T stage	1.841	1.297	2.612	0.001	1.933	1.342	2.784	< 0.001
Lymph node involvement	1.613	0.775	3.358	0.201	1.708	0.823	3.546	0.151
Grade	0.584	0.069	4.149	0.589	0.461	0.051	5.286	0.685
De Ritis ratio (Low vs. high)	2.755	1.214	6.249	0.015				
De Ritis ratio (Continuous)					1.848	1.239	2.755	0.003
Multivariate Cox proportional analysis of overall death								
Age (< 70 vs. ≥70)	0.999	0.963	1.037	0.956	1.004	0.969	1.041	0.807
Gender (male vs. female)	0.471	0.161	1.377	0.169	0.337	0.088	1.285	0.111
BMI	0.972	0.858	1.100	0.651	0.952	0.840	1.078	0.436
Pathologic T stage	1.921	1.368	2.698	< 0.001	2.017	1.415	2.876	< 0.001
Lymph node involvement	1.572	0.778	3.176	0.207	1.694	0.842	3.409	0.139
Grade	0.326	0.065	5.372	0.538	0.556	0.064	4.718	0.538
De Ritis ratio (Low vs. high)	2.761	1.257	6.067	0.011				
De Ritis ratio (Continuous)					1.860	1.262	2.743	0.002

*HR* hazard ratio, *CI* confidence interval, *BMI* body mass index

## Discussion

BC most commonly affects elderly individuals and those with significant comorbidities and an impaired performance status [23]. RC remains the gold standard of treatment in patients with local MIBC and in some cases of NMIBC [18]. However, despite these aggressive local approaches, long-term prognosis remains poor due to disease recurrence accompanied by local and/or distant metastasis [24]. These poor outcomes suggest a need for ongoing risk stratification and appropriate selection of multimodal treatment approaches, such as chemotherapy in neoadjuvant or adjuvant settings. With recent advances in techniques, an extremely large number of new prognostic markers have been identified [25]. New biomarkers predictive of outcomes would help clinicians provide risk stratification for patients and serve as prognostic indicators for individual patients [26]. For clinical practice purposes, a potential prognosticator would generally have great potential if it could be easily and inexpensively determined by routine measures.

In this study, we determined that an elevated De Ritis ratio showed a significant association with adverse outcomes in patients with BC who underwent RC. Patients with elevated De Ritis ratios showed significantly inferior survival outcomes in terms of MFS, CSS, and OS after RC in patients with BC. Moreover, elevated De Ritis ratios were found to be significant independent predictors of metastasis, cancer-related death, and overall death. To our knowledge, this study is the first to identify the De Ritis ratio as a novel significant prognostic biomarker in BC. Because the De Ritis ratio may be inexpensively and reproducibly measured, it might become a promising tool in the management of BC.

The amino transaminase enzymes (AST and ALT) which are strongly involved in cellular metabolism and cancer cell turnover, represent easily measurable potential blood-based biomarkers [12]. Amino transaminases are widely used to determine liver function in clinical care. Moreover, amino transaminases are used to identify liver diseases such as viral hepatitis and alcohol abuse. De Ritis et al. were the first to introduce the AST/ALT ratio as a useful indicator for differentiating the etiology of acute hepatitis [8]. Previous studies of certain types of cancer indicate that different levels of these enzymes are associated with patient prognoses [11–13, 27, 28].

For instance, the results from Tan et al. showed that AST/ALT ratio over 2.0 was an independent prognosticator of poor survival in patients with distal cholangiocarcinoma, [28], and Bezan et al. represented that preoperatively assessed De Ritis ratio (> 1.26) was significantly associated with the clinical course of patients with non-metastatic RCC [12]. Rawson and Peto retrospectively analyzed 3873 patients with small cell lung cancer and concluded that AST was a significant prognostic factor [27]. Lindmark et al. also reported that AST and ALT were significantly associated with patient survival after analyzing 212 patients with colorectal cancer [9]. Stocken et al. analyzed the data of 653 patients with advanced pancreatic cancer from 2 randomized studies. Stockon et al. also concluded that AST was independently related to CSS [11]. An additional study conducted by Kiba et al. reported that high AST and lactate dehydrogenase levels were significantly associated with inferior OS in patients who received bortezomib and dexamethasone chemotherapy for multiple myeloma [10]. Moreover, Nishikawa et al. first reported that the De Ritis ratio was a significant prognostic biomarker in UTUC [13]. They conducted a retrospective multivariate analysis of 109 patients and concluded that the De Ritis ratio, pathological stage, lymph node metastasis, and tumor grade were independent predictors of extravesical recurrence-free survival.

An enormous rise in glycolysis and glucose uptake was needed in tumorigenesis and cancer progression. Actually, the formation of abundant pyruvate and lactate is the common characteristics of tumor cells. Warburg discovered this event [29]. Although there is oxygen, the increase in the velocity of glucose delivery is accompanied by enhanced glycolysis. Increased glycolysis is known to be linked to several alterations in mitochondrial activity. Furthermore, increased glycolysis is also associated with nicotinamide adenine dinucleotide-related enzyme activity and glucose transporter activity [30].

Moreover, the malate-aspartate shuttle pathway has AST as a main component. Consequently, tumor metabolism might be associated with De Ritis ratio in several glucose using cancers. In addition, BC is noted as a glucose-reliant malignant tumor [14, 15, 30]. The uptake of glucose by BC cells via using fluorescence microscopy was explored by Whyard et al. [30]. They found that noteworthy changes in glucose consumption between normal urothelium and tumor cells. In this study, increased lactate production amplified the pyruvate synthesis and the concentration of glutamine was elevated. The Warburg effect has all of these features. Based on these results and our data from the present study, it is likely that De Ritis ratio was linked to BC. However, the precise mechanism for revealing the relationship between increased De Ritis and poor prognosis of BC patients has not been established.

We would like to emphasize several drawbacks of this study. Initially, this was a retrospective study with a comparatively small number of patients, and the follow-up period was relatively short. Second, the existence of undetected liver pathologic conditions that can influence the serum levels of AST or ALT might distort the De Ritis ratio, although we excluded the patients with chronic liver disease (hepatitis, liver cirrhosis, and severe fatty liver disease) including hepatitis B or C virus carriers In addition, we did not evaluate the smoking history of the patients. Smoking is a significant risk factor for BC and plays an important role in BC progression; however, it was very difficult to obtain exact information on patient smoking. A self-completed questionnaire about smoking may have introduced recall bias. Some studies in Korea about the association between *BC* and smoking history showed discordant results. Lastly, we included only Korean population in our cohort. Additionally, studies of other ethnic groups are required before our results can be applied universally.

## Conclusions

This study is the first of its kind to investigate the effect of a preoperative assessment of the De Ritis ratio. Our study was also the first to include the prognosis of patients with BC who underwent RC. An elevated De Ritis ratio was found to significantly increase the risk of metastasis, cancer-specific death, and general mortality after undergoing RC for BC. The De Ritis ratio should be regarded as an important tool to be used in counseling patients regarding expected outcomes.

## Abbreviations

ALT: Alanine aminotransferase
AST: Aspartate aminotransferase
BC: Bladder cancer
BCG: Bacillus Calmette–Guérin
CI: Confidence interval
CSS: Cancer-specific survival
HR: Hazard ratio
MFS: Metastasis-free survival
MIBC: Muscle-invasive bladder cancer
NMIBC: Non-muscle-invasive bladder cancer
OS: Overall survival
RC: Radical cystectomy
RCC: Renal cell carcinoma
TUR-BT: Transurethral resection of bladder tumor
UTUC: Upper tract urothelial cancer
